# Early-Life Dam-Calf Contact and Grazing Experience Influence Post-Weaning Behavior and Herbage Selection of Dairy Calves in the Short Term

**DOI:** 10.3389/fvets.2020.600949

**Published:** 2020-12-07

**Authors:** Alessandra Nicolao, Mauro Coppa, Matthieu Bouchon, Enrico Sturaro, Dominique Pomiès, Bruno Martin, Madeline Koczura

**Affiliations:** ^1^Université Clermont Auvergne, INRAE, VetAgro Sup, UMR Herbivores, Saint-Genès-Champanelle, France; ^2^DAFNAE, University of Padova, Legnaro, Italy; ^3^Independent Researcher at Université Clermont Auvergne, INRAE, VetAgro Sup, UMR Herbivores, Saint-Genès-Champanelle, France; ^4^INRAE, UE Herbipôle, Saint-Genès-Champanelle, France

**Keywords:** grazing behavior, dairy calves, grazing experience, dam-calf contact, post-weaning, social interactions, first grazing

## Abstract

Rearing dairy calves with their mothers could teach them how to graze, optimizing grass use, and improving their welfare and performance. We tested the short-term effects of dam-calf contact experience on grazing and social behavior of weaned calves, monitored over seven days for their first post-weaning grazing experience. “Dam” (D) calves were reared and grazed with their mothers until weaning. “Mixed” calves (M) were separated from their mothers after 4 ± 0.5 weeks, they experienced dam-calf contact, but not grazing. “Standard” (S) calves had never experienced either dam-calf contact (separated at birth) or grazing. Each group grazed an equivalent pasture plot offering heterogeneous herbage. Scan sampling of calves' activities was performed every 5 min, 6 h per day, on Days 0, 1, 2, 3, and 7. Daily, the time when calves started grazing after introduction to pasture, and the number and duration of their grazing cycles were measured. Daily activities were differentiated into ingestion, rumination, and idling. The proportion of time that calves spent grouped with other individuals or isolated, and standing or lying were recorded. When grazing, their bites were characterized by botanical family group, height of the selected bite and vegetation status. Individual average daily gains from the 2-week periods before and after grazing were calculated, and were equivalent between groups (313 ± 71 g/d). On Day 0, D-calves started grazing immediately (1 ± 4.1 min), unlike M- and S-calves (39 ± 4.1 and 23 ± 4.1 min), and D-calves grazed patches of dry grass 21.7 times less than M-calves and 16.9 times less than S-calves. Dry herbage patch preference and grazing start time differences disappeared on Day 1. Calves spent the same time ingesting and idling, but M-calves spent on average 1.6 times less ruminating than D- or S-calves. The D-calves showed grazing behavior similar to that of adult cows, selecting grasses throughout pasture utilization, although legumes and forbs were present in the grazed layer. On the contrary, M- and S-calves did not express any specific preference. The S-calves spent more time isolated but had more positive reciprocal interactions than the calves in the other groups.

## Introduction

Maximizing production while reducing costs and labor are the main aims of modern dairy systems. This trend often results in an intensification of farming practices, which weakens societal acceptance of dairy production systems (1). Consumers are taking ever greater interest in how their food is produced, and are increasingly aware of environmental issues and animal welfare (2, 3). In dairy production, the most common welfare concerns are the separation of calves from their dams (4) and restricted access to pasture for animals in intensive systems (5). Pasture for dairy cattle offers several advantages for animal welfare and health, such as expression of natural behavior and possible reduction of lameness and claw disorders (6–8) or increased movement with positive effects on longevity (9). Grazing systems also reduce management and feeding costs for the farmer (10, 11). In commercial dairy farms, calves are usually separated from their dams close to birth, and rarely experience grazing during their early lives (12). In France, 60 % of dairy farms use seasonal batch calving during autumn and winter, in order to turn out animals to pasture in the following spring (13). Then, calves and heifers usually graze from spring to autumn, before their first year of age, but only 2% of dairy farms turn out calves to pasture before 6 months of age (13). This strategy allows the synchronization of the peak of herbage growth and the peak of lactation of dairy cows, with fresh herbage covering a large part of their nutritional requirements (14). At the same time, calves have also grown and matured sufficiently and are able to be moved to pasture.

Le Cozler et al. (13) reported that only 4% of farmers keep calves with their dam at later than 24 h, but this practice is increasingly used. Michaud et al. (15) investigated farms using a suckling practice in France (Massif Central, East and West of France), and found that 62 farms out of 102 kept calves with their dam or with a foster cow between 1 and 60 days of age. The presence of the dam in the early stages of a calf's life can have positive effects on its social interactions, feeding behavior, and growth (16–18). The dam is the primary social model and plays an important role in the acquisition of foraging behavior and feed selection (19, 20). Pullin et al. (20) found that lambs grazing with their dam spent more time foraging, were more active, developed long-term feed preferences and learned aversion to toxic feed more effectively than lambs grazing alone. Young animals learn by emulation of social models or by trial and error, although in most cases this last is less efficient (21). Calves usually are neophobic: they tend to choose feed and places they already know, so that individual learning in a new environment takes more time than learning by social models (22, 23). Lopes et al. (24) observed that heifers with early grazing experience, compared to inexperienced heifers, affected grazing behavior and milk production only in the first days on pasture, but showed that the animals would generally adapt to a new environment and a novel feed easily, especially during their first year of life. Dairy calves that have learned to graze with their dam might therefore more efficiently recognize herbage quality and select specific patches when turned out to pasture after weaning, compared to calves that never grazed before. However, it is unclear whether this advantage holds only in the first grazing day or is more persistent.

In the present study, the following hypotheses were tested, comparing three groups of calves with contrasting rearing experience on their first grazing days after weaning. We expected calves that had experienced dam-calf contact and grazing in their early life to show grazing and probably social behavior that was different from that of inexperienced calves, and more typical of adult dairy cows. The longer dam-calf contact lasted (a few weeks or until weaning), the greater would be the expected differences in calves' social behavior. The present study also evaluated the persistence of the expected differences in grazing or social behavior in the short term after weaning.

## Materials and Methods

### Experimental Design

The experiment was performed in 2019 at the INRAE experimental farm of Marcenat (DOI: https://doi.org/10.15454/1.5572318050509348E12), located in the Massif Central (45°15′N, 2°55′E; 1150 m a.s.l.). All animal-related procedures were carried out in accordance with the guidelines for animal research of the French Ministry of Agriculture and all other applicable national and European regulations for experimentation with animals (https://www.recherche-animale.org/sites/default/files/charte_nationale_portant_sur_l_ethique_de_l_experimentation_animale_243579.pdf). The experiment started February 12. The early grazing period started July 22 and ended July 29. Three breed-balanced groups of eight dairy calves (Holstein and Montbéliarde) with different experience backgrounds were compared (Table 1): a group of “Standard” calves (S) that had been separated at birth from their dam and had never experienced grazing, a group of “Dam” calves (D) that had been reared and grazed with their dam until weaning, and a group of “Mixed” calves (M) that had been separated from their dam at 4 ± 0.5 weeks of age and had never experienced grazing. All calves were weaned at age of 10.9 ± 1.1 weeks. Before weaning, D-calves were housed separately from their dams at night and had free access to the dam cowshed during the day. Starting from May 5, when the calves were 4.6 ± 3.2 weeks old, the day cowshed access was replaced by free access to pasture with dams. The M-calves, until age 4.0 ± 0.5 weeks, were reared in the same way, except that they had no access to pasture. From this age until weaning, they were reared like S-calves, i.e., in separate housing and fed bulk milk with an automatic milk dispenser. D- and M-calves were reunited with their dams after morning milking at 9:00 a.m. and separated before evening milking at 3:30 p.m. At weaning, all calves were moved to a new pen, with one pen for each group to prevent mixing. In this pen, calves ingested 0.5 kg/d/calf of hay and 2.0 kg/d/calf of concentrate (Startivo, Centraliment, 15006 Aurillac). Hay was distributed in the evening with no refusal left in the morning. Concentrate was distributed half in the morning and half in the evening, until the end of the study. After the last weaning, all calves spent at least six days indoors all together to allow the latest weaned calves to adapt to the new conditions. At the beginning of the grazing period (week 15), D-, M- and S-calves were, respectively, 14.9 ± 3.2, 16.1 ± 2.8 and 15.3 ± 3.6 weeks old and weighed 131 ± 18.3 kg, 123 ± 17.4 kg, and 128 ± 23.5 kg respectively, on average. They had been weaned for 30 ± 22, 33 ± 20 and 33 ± 24 days, respectively. Calves were turned out to pasture on July 22 (Day 0), from 9:00 a.m. to 5:00 p.m.

**Table 1:** Feeding plan (milk, concentrate, and hay) of the three groups of calves (Standard, Dam, Mixed) during the first 15 weeks of age.

**Group**	**Week**	**1**	**2**	**3**	**4**	**5**	**6**	**7**	**8**	**9**	**10**	**11**.	**15**
Standard	Milk^1^ (kg/d)	6.0	7.0	9.0	10.0	10.0	10.0	9.0	7.0	5.0	3.0	Weaning	Start grazing
	Concentrate^2^ (kg/d)	0	0	0.2	0.4	0.6	0.9	1.2	1.5	1.8	2.0	2.0	2.0
	Hay^3^	0	*ad libitum*									*ad libitum*	0.5
Dam	Suckling period	24 h/24 h	Between morning and evening milkings (=during the day)									Weaning	Start grazing
	Concentrate (kg/d)	0	*ad libitum*									2.0	2.0
	Hay	0	*ad libitum*									*ad libitum*	0.5
	Pasture with dams	/	/			*During the day*						/	
Mixed	Suckling period	24 h/24 h	During the day		10.0	10.0	10.0	9.0	7.0	5.0	3.0	Weaning	Start grazing
	Concentrate (kg/d)	0	*ad libitum*		0.4	0.6	0.9	1.2	1.5	1.8	2.0	2.0	2.0
	Hay	0	*ad libitum*		*ad libitum*							*ad libitum*	0.5

1 *bulk milk distributed individually by automatic feeder*.

2 *first age concentrate distributed individually by automatic feeder (Standard group and Mixed group after separation from the dam) or in collective bucket (Dam group and Mixed group before separation from the dam)*.

3 *permanent grassland hay (first cut) distributed in a rack*.

### Characteristics of the Experimental Plot

Calves grazed a permanent pasture divided into three equivalent neighboring 0.15 ha plots. No close visual contact was allowed between the three groups of animals, by fencing the plots so that they were at least 15 m apart. To encourage grazing selection for all three groups of calves, the whole plot was strip-mown 28 days before the grazing period started. At Day 0, plots were thereby composed of alternate 3 m strips dominated by mature vegetation and vegetative regrowth. The botanical composition of the whole pasture was determined using the vertical point-quadrat method from Daget and Poissonet (25). The pasture was dominated by *Lolium perenne* (39.0%)*, Agrostis tenuis* (15.0%), and *Trifolium repens* (13.5%). *Rumex obtusifolius* was also present (3.1%). At the beginning of the experiment, three 10 cm × 3 m grass samples were collected on each plot, perpendicularly to the mown and unmown strips, equally harvesting the same length from both. They were oven-dried at 60°C for 72 h and analyzed for proximate composition as described by Coppa et al. (26) (Table 2).

**Table 2:** Characteristics of vegetation offered on the experimental plots (mean ± standard deviation).

**Plot characteristics**	**Dam**	**Mixed**	**Standard**
***Patch type (%) and description***			
Dry (≥70% dead material)	15.5 ± 4.1	16.0 ± 2.1	13.3 ± 2.5
Green (< 70% dead material)	84.5 ± 7.2	84.0 ± 4.1	86.7 ± 6.0
Grasses (≥ 70% grasses)	65.1 ± 9.7	69.1 ± 6.6	64.2 ± 7.3
Legumes (≥ 30% legumes)	17.1 ± 4.2	13.6 ± 2.0	13.3 ± 3.4
Forbs (≥ 30% forbs)	17.8 ± 3.6	17.3 ± 1.4	22.5 ±4.8
Tall (≥ 25 cm)	51.2 ± 7.8	48.1 ± 4.5	53.3 ± 7.6
Intermediate (7 cm ≤ x <25 cm)	33.3 ± 5.7	35.8 ± 4.1	32.5 ± 5.1
Short (< 7 cm)	15.5 ± 5.2	16.0 ± 2.6	14.2 ± 3.1
***Composition and nutritional value***			
Dry matter *(g/kg)*,	32.5 ± 3.3	28.3 ± 5.9	31.6 ± 2.5
Organic matter digestibility *(g/kg DM)*	67.2 ± 2.8	67.0 ± 3.8	66.0 ± 1.7
NDF *(g/kg DM)*	53.9 ± 4.4	53.8 ± 1.2	53.9 ± 1.8
ADF *(g/kg DM)*	27.6 ± 1.6	28.2 ± 1.0	28.5 ±1.9
Crude protein *(g/kg DM)*	12.0 ± 1.6	12.2 ± 2.7	10.6 ±1.8

### Observations and Behavior Measurements

Calves were weighed once a week, and individual average daily gain (ADG) from the 2-week periods before and after the grazing period started was calculated.

**Figure 1:**
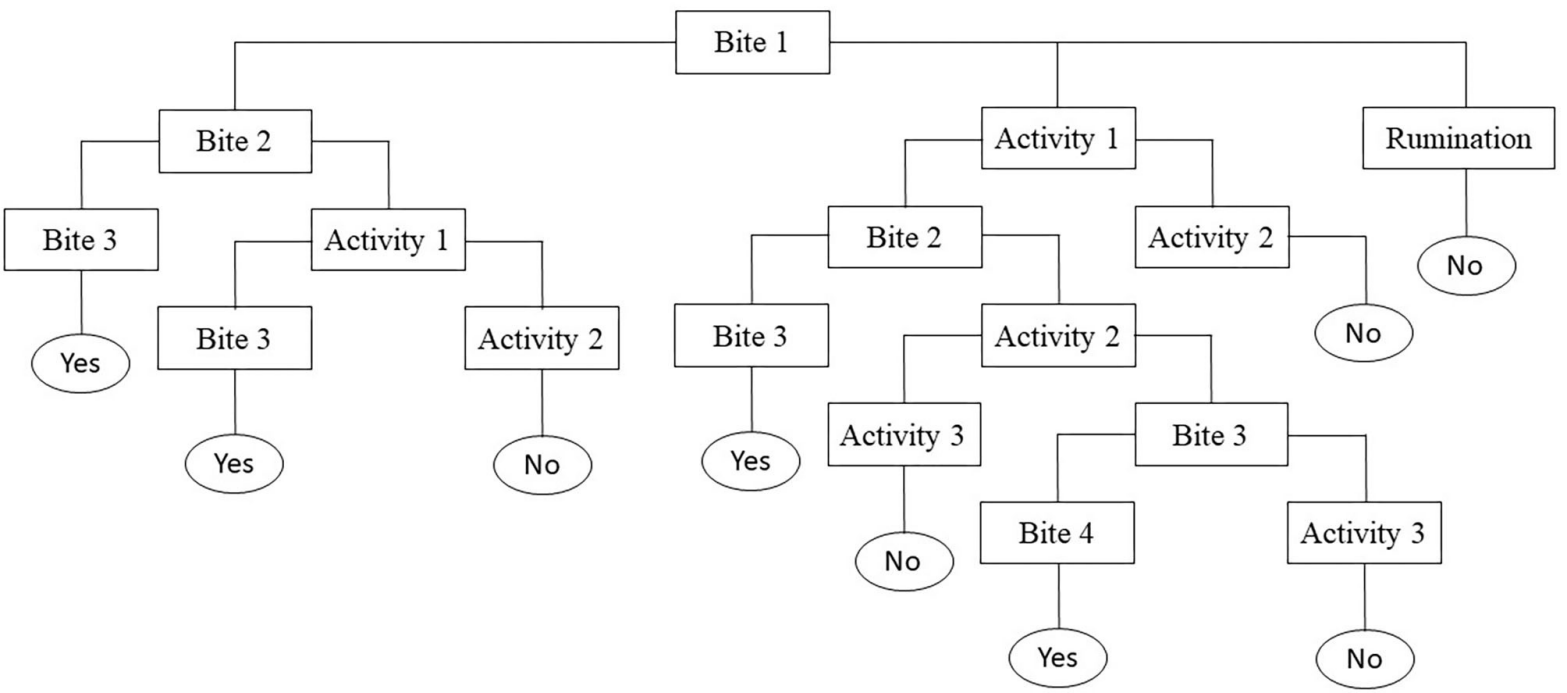
Flowchart of the conceptual scheme used during observations to tell whether calves started grazing at Bite 1 (Yes = at least three bites over four observations; No = flowchart restart to the next observation).

**Figure 2:**
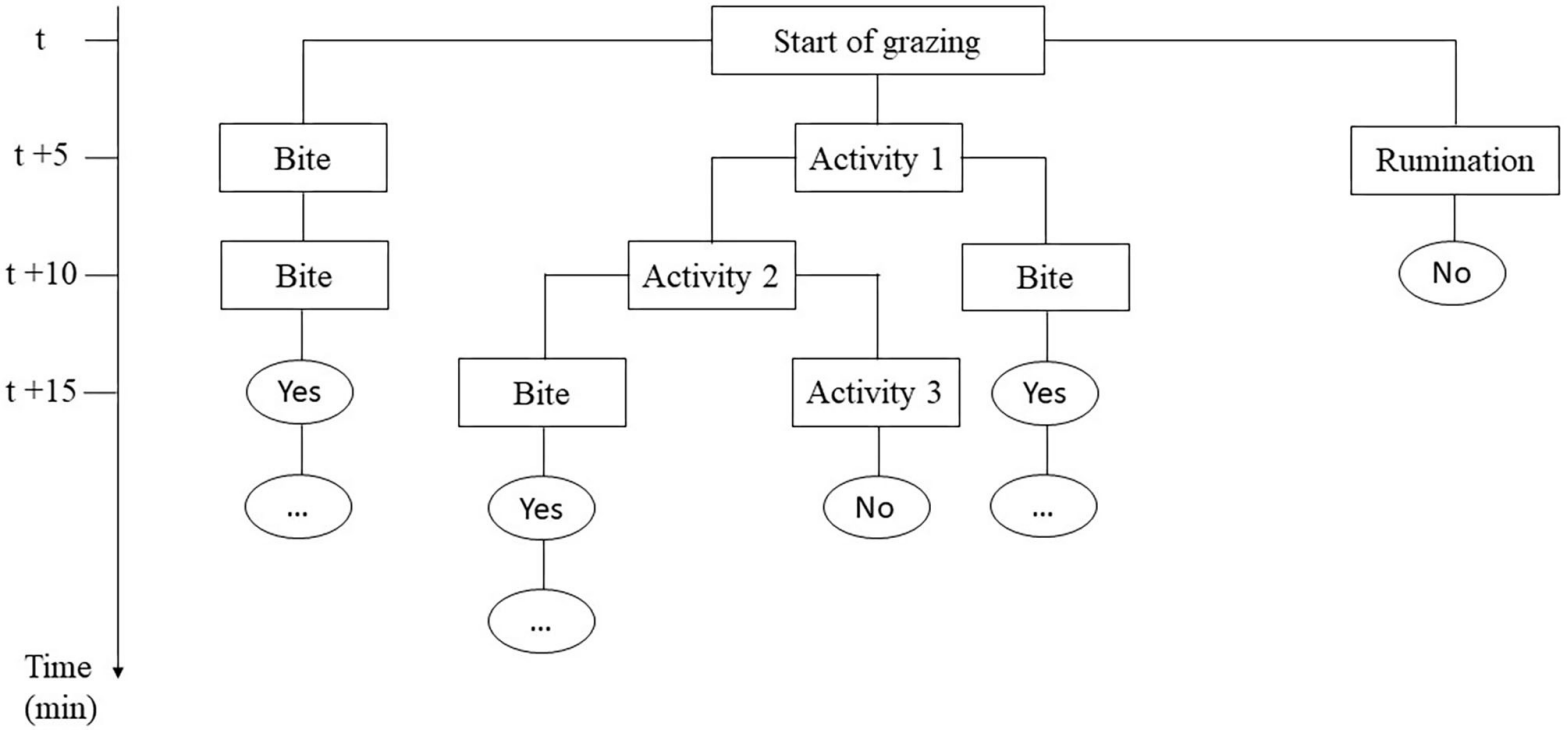
Flowchart of the conceptual scheme used during observations to tell whether a grazing cycle was established (Yes or No) and measure its duration.

Individual daily activities and behavior were observed by scan sampling at 5-min intervals (27, 28) on the day the calves encountered the pasture for the first time (Day 0), the next three days (Day 1, Day 2, Day 3) and one week later (Day 7), focusing on the first days as most of the differences were expected here (24). On each plot, four calves were randomly assigned to two observers for 6 h per day (9:00–12:00 a.m. and 2:00–5:00 p.m.). For observations, calves were always identified by the same numbers painted on their back. At the end of the afternoon, the calves went back indoors for the night where they were fed with hay and concentrate (Startivo, Centraliment, 15006 Aurillac). Observers, randomly assigned to a group of calves, changed experimental group between each morning and afternoon. Each day, the time taken by calves to start grazing was measured. A calf was considered to have started grazing if it was observed taking a bite in at least three out of four successive observations (29), following the flowchart in Figure 1. From the time the calf started grazing, the grazing cycle lasted until it showed more than three other successive activities (i.e., it stopped grazing for at least 15 min), according to Manzocchi et al. (30). The duration of a grazing cycle and the number of grazing cycles, as just described, were calculated following the flowchart in Figure 2. Daily activities were then differentiated into three groups: ingestion (grazing and drinking water), rumination, and idling. The latter comprised four subcategories: resting (observation, sleep, self-grooming), positive interactions (licking, sniffing, head play), negative interactions (head-butting, pushing, fighting) and *ad hoc* activities (walking, exploring, stereotypies, vocalizing) (Table 3). The daily proportion of ingestion, rumination and idling time was calculated as a percentage of the total daily observations. The daily proportion of resting time, socializing time and *ad hoc* activities was calculated as a percentage of the idling activities. Each time one of the activities was recorded, observers also indicated whether the calf was grouped with other calves or isolated, i.e., at least 3 m away from other calves, and whether it was standing or lying. The daily proportions of time spent grouped and standing were calculated over the total number of observations of the day. When calves were grazing, their bites were characterized by botanical group (grasses, legumes and forbs), the height of the selected bite (tall, intermediate, short vegetation) and the vegetation status (“dry” or “green”), according to Koczura et al. (31). Briefly, patches were characterized according to the visually estimated proportion of dry senescent herbage, of botanical family groups and of their height (26, 32). A patch was coded as “dry” if the dry senescent vegetation represented more than 70 % of the bite, as “green” if it was <70%; as dominated by “grasses” if the bite contained more than 70% of grasses, by legumes or forbs if they represented more than 30%; tall if herbage height was ≥ 25 cm, and small if it was ≤ 7 cm, as detailed in Table 2. Observers were able to get close to calves due to their adaptation to human presence achieved during the pre-weaning experiment. When calves ingested forbs, observers reported whether or not they selected *Rumex* thanks to a binary variable (1 = the calf tried to eat *Rumex* at least one time in the observation day). The daily proportion of vegetation type ingested by calves was calculated as a percentage of observations comprising the vegetation type compared to the total number of grazing observations of the day.

**Table 3:** Description of daily activities recorded by scan-sampling differentiated in four subcategories.

**Daily activity**	**Subcategory**	**Behavior type**	**Description**
Ingestion			*Grazing and drinking water*
Rumination			*Ruminating*
Idling	Resting	Observation	*Standing or lying, without sleeping*
		Sleep	*Sleeping*
		Self-grooming	*Self-licking, rubbing, defecating and urinating*
	Positive interaction	Licking	*Licking another calf's head or body*
		Sniffing	*Sniffing another calf's head or body*
		Head play	*Rubbing the head against the head of another calf*
	Negative interaction	Head-butting	*Pushing the head against the head of another calf*
		Pushing	*Pushing the head against the body of another calf*
		Fighting	*Two calves pushing each other*
	*Ad hoc* activities	Walking	*Walking*
		Exploring	*Sniffing the floor, sniffing/ licking objects, discovering the environment*
		Stereotypies	*Cross-suckling, tongue rolling and repeatedly sniffing/licking objects*
		Vocalizing	*Mooing punctually and/or repeated*

The weather was exceptionally hot on the afternoons of Day 2 and Day 3. The average maximum daily temperature during these afternoons was 31.2°C, whereas between 2000 and 2019, the average maximum temperature in July was 21.6°C (INRAE CLIMATIK 2.1.5, Marcenat weather station). Behavior observations at pasture were therefore made throughout the day on Day 0, Day 1, and Day 7, but only in the morning on Day 2 and Day 3. The daily ingestion, rumination and idling activities, together with the number and duration of grazing cycles, were accordingly calculated only for Day 0, Day 1, and Day 7, as the morning alone was not considered representative of the ingestion and rumination cycles of a whole day. On the other hand, the characterization of grazed bites and time needed to start grazing were calculated on mornings only for all days, the numbers of bites observed during the morning being considered sufficient and representative to express preference, as differences between morning and afternoon on those days were equivalent.

### Statistical Analysis

Daily activities and grazing cycles were analyzed with a repeated MIXED model on SAS 9.4 software (SAS Institute Inc., Cary, NC, USA). Group (Dam, Mixed or Standard), day (only 0, 1, or 7) and their interaction were included as fixed effects. The individual calf was considered as the subject of repetition, with day being the repeated factor. We used a compound symmetry covariance structure. Time to start grazing and herbage selection were analyzed with the same model, except that the day effect included all days. Average daily gain was analyzed with a similar repeated model, which included group, period (before or after pasture) and their interaction as fixed effects, calf as subject, and period as repeated factor. In this last model, the number of days since each calf had been weaned was used as a covariate. The effect of the age and BW of calves were tested as covariates as well, but were found to be non-significant, and so were not finally included in the model. For all data, normality of residuals was checked using the Shapiro-Wilk test. The frequency of times calves tried at least 1 time to include *Rumex* in their bites was compared between groups using a Chi^2^ test. Significance was set at *p* < 0.05.

## Results

### ADG Before and After Grazing

During the 2 weeks before start of grazing, the ADG of D-, M- and S-calves did not differ significantly (*p* = 0.177), at 285, 355, and 480 g/d, respectively. During the following 2 weeks it increased by 313 g/d on average for all the groups.

### Effect of Early Dam-Calf Contact and Grazing Experience on Calves' Daily Activities

**Figure 3:**
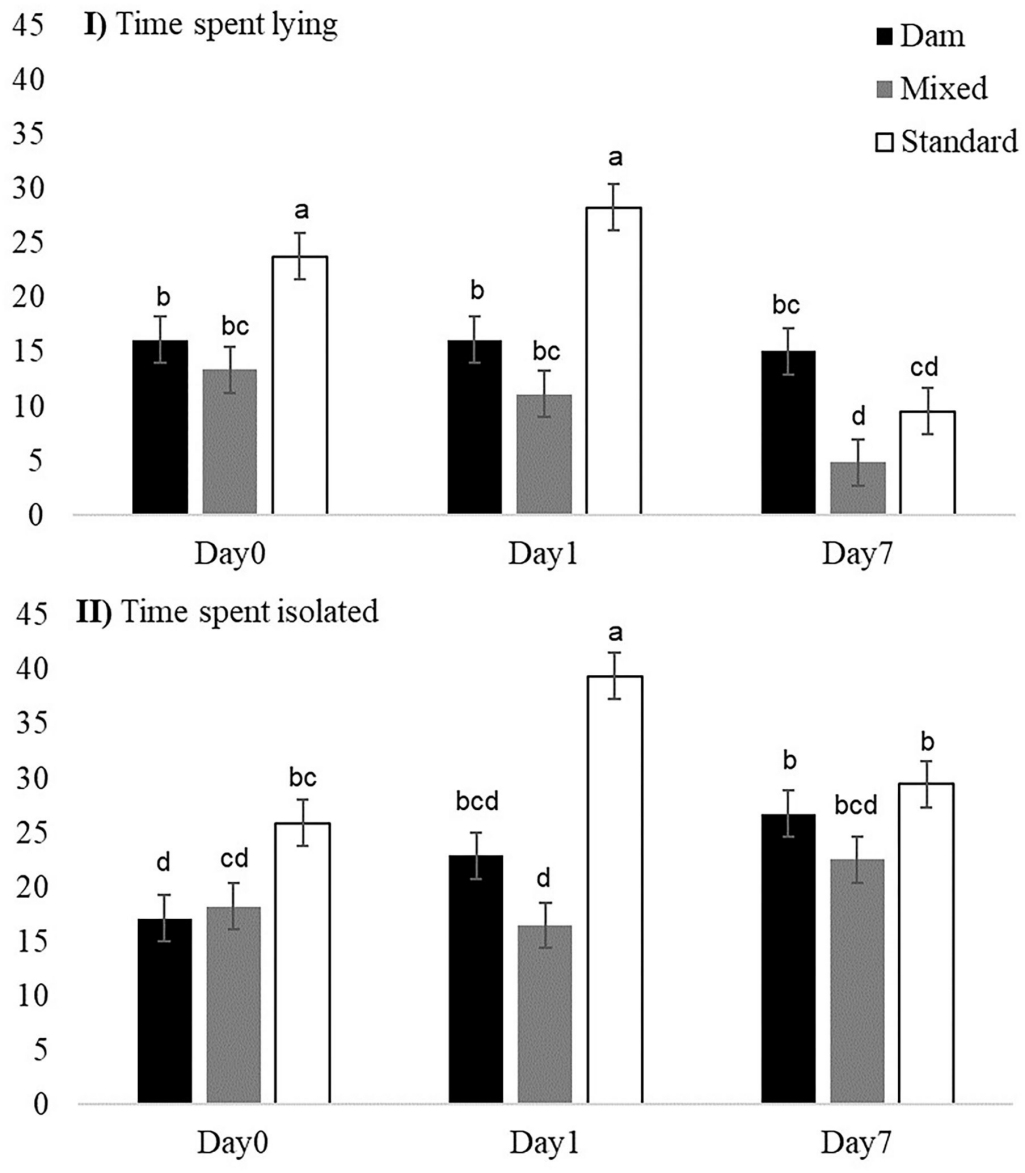
Effect of early dam-calf contact and grazing experience on **(I)** daily time spent lying (%) and **(II)** isolated (%) by calves on Day 0, Day 1, and Day 7 at pasture. Bars are standard errors. ^a−d^Means within a variable with different superscript letters differ at *p* < 0.05.

Once turned out to pasture, the daily activities of the three groups of weaned dairy calves did not strongly differ (interaction groups × day non-significant). Overall, during Days 0, 1, and 7, calves in the three groups spent almost the same time ingesting (*p* = 0.081, on Day 1 M-calves tended to spend less time ingesting than D- and S-calves) and the same time idling (Table 4), but M-calves spent on average less time ruminating than D- and S-calves (1.54 times less). During idling activities, D-calves spent more time resting than M- or S-calves (1.16 and 1.14 times more, respectively), and M-calves spent more time in *ad hoc* activities than D-calves (1.24 times more). The S-calves had more positive social interactions than the calves in the other two groups. On Day 0 and Day 1, S-calves spent more time lying than D- or M-calves (Figure 3I). On Day 0 and Day 1 they spent more time isolated than calves in the other two groups (Figure 3II).

**Table 4:** Effect of early dam-calf contact and grazing experience on post-weaning daily activities and grazing cycles of dairy calves (Day 0, 1 and 7 after start of grazing).

**Item**	**Dam**	**Mixed**	**Standard**	**SEM**	**Group**	**Day**	**Group × day**
**Daily activities (% of daily total observations)**							
Ingestion time	55.8	58.3	58.2	1.88	ns	^**^	^*†*^
Rumination time	11.0^a^	6.9^b^	10.2^a^	1.01	^*^	^*^	ns
Idling time	33.2	34.8	31.7	1.88	ns	^***^	ns
Grazing cycles (by day)							
Duration (min)	57.8	55.9	55.6	4.97	ns	^*†*^	ns
Number	3.1	3.5	3.2	0.21	ns	^*^	ns
**Idling activities (% of daily idling observations)**							
Resting time^1^	64.6^a^	55.4^b^	56.5^b^	2.55	^*^	^***^	ns
*Ad hoc* activities^2^	30.2^b^	37.3^a^	35.4^ab^	1.89	^*^	^***^	ns
Positive interactions^3^	1.6^b^	0.7^b^	3.3^a^	0.51	^**^	ns	ns
Negative interactions^4^	3.7	6.5	4.8	1.07	ns	^**^	ns
**Proportion of time (% of daily observations) spent:**							
Lying	15.8^b^	9.8^c^	20.5^a^	1.60	^***^	^***^	^***^
Isolated	22.3^b^	19.1^b^	31.6^a^	2.07	^***^	^**^	^***^

*** p < 0.001;

** p < 0.01;

* p < 0.05;

† *p < 0.10; ns p ≥ 0.10*.

a−c *Means within a variable with different superscript letters differ at p < 0.05*.

1 *Resting time: observation, sleeping, self-grooming*.

2 *Ad hoc activities: walking, exploring, stereotypies, vocalizing*.

3 *Positive interaction: licking, sniffing, head play*.

4 *Negative interaction: head-butting, pushing, fighting*.

### Effect of Early Dam-Calf Contact and Grazing Experience on Calves' Herbage Selection

**Figure 4:**
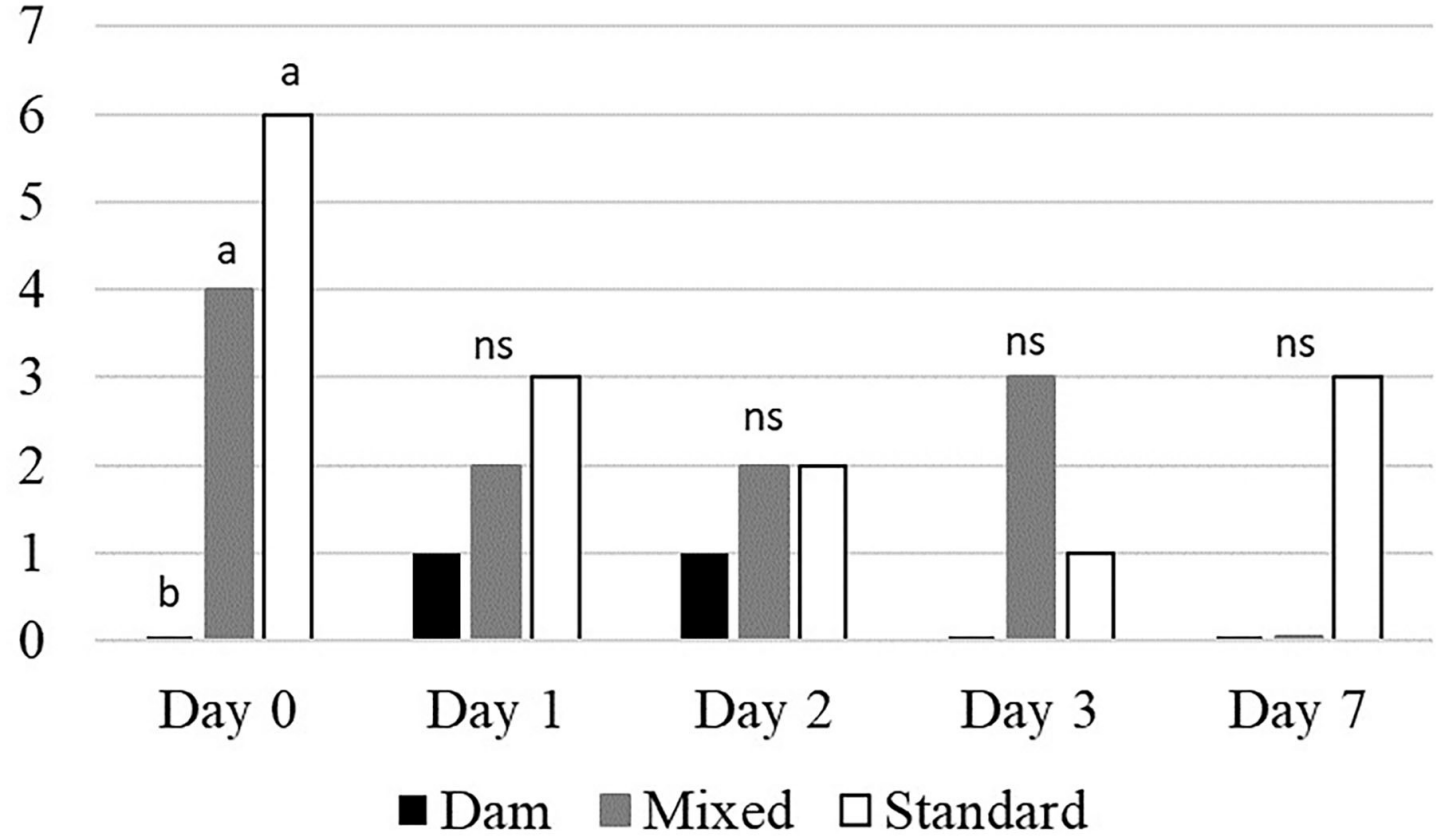
Overall number of calves that tasted *Rumex* each day (*n* = 8 calves × 3 groups). ^a−b^Means with different superscript differ at *p* < 0.05, Khi^2^ test; *ns*, not significant.

When moved to pasture, D-calves started grazing immediately (Table 5), whereas it took S-calves 23 ± 4.1 min to actively start to graze. The M-calves needed a further 20 min. On Day 0, the herbage selection was different between groups: D-calves grazed “dry” patches 21.71 times less than M-calves and 16.90 times less than S-calves. On Day 0, no differences between groups were observed for botanical composition and height, except for forbs: on that day, S-calves grazed 13.73 times more forbs than M-calves and 3.89 times more than D-calves. On Day 1, Day 3, and Day 7 all three groups of calves started grazing 5 ± 2.8 min after arriving on pasture, whereas M- and S-calves started grazing 15 ± 0.7 min after D-calves on Day 2. From Day 1, M- and S-calves reduced their proportion of “dry” patches to meet that of D-calves, with no longer any significant differences between groups. Overall, we found that the proportion of tall vegetation in the bites decreased from Day 1 to Day 7 and conversely that the proportion of short vegetation increased in the bites from Day 1 to Day 7. On Day 2 and Day 3, M-calves showed a higher proportion of intermediate vegetation than D- and S-calves (2.07 and 3.03 times more, on average). D-calves continuously maintained stable the proportion of grasses in their bites throughout the plot utilization, while M- and S-calves decreased their proportion over time (0.75 times less from Day 0 to Day 7, on average), increasing in parallel those of legumes and forbs (7.57 and 4.46 more times on average, respectively). On Day 0, none of D-calves grazed *Rumex*, on the contrary to M- and S-calves (4 and 6 calves, respectively) (Figure 4). This difference disappeared in the following days, already on Day 1.

**Table 5:** Effect of early dam-calf contact and grazing experience on time to start grazing after introduction to pasture and characteristics of selected bites by dairy calves.

**Item**	**Group**	**Day**					**SEM**	**Group**	**Day**	**Group × day**
		**0**	**1**	**2**	**3**	**7**				
Time to start grazing (min)	Dam	1^e^	6^e^	3^e^	2^e^	4^e^	4.13	^***^	^***^	^***^
	Mixed	39^a^	4^e^	18^bcd^	11^cde^	7^de^				
	Standard	23^b^	4^e^	19^bc^	6^e^	2^e^				
Herbage selection (% of ingestion observations)										
Green	Dam	97.9^a^	100^a^	97.2^a^	100^a^	98.1^a^	0.03	^***^	^***^	^***^
	Mixed	54.5^c^	98.0^a^	99.2^a^	98.5^a^	95.2^a^				
	Standard	64.5^b^	94.6^a^	92.8^a^	98.3^a^	99.5^a^				
Dry	Dam	2.1^c^	0^c^	2.8^c^	0^c^	1.9^c^	0.03	^***^	^***^	^***^
	Mixed	45.6^a^	2.0^c^	0.8^c^	1.5^c^	4.8^c^				
	Standard	35.5^b^	5.4^c^	7.2^c^	1.7^c^	0.5^c^				
Grasses	Dam	86.4^abc^	75.6^cd^	81.3^abc^	91.9^a^	87.1^abc^	0.04	^***^	^***^	^***^
	Mixed	87.8^ab^	86.8^abc^	59.5^ef^	52.7^f^	66.6^de^				
	Standard	77.8^bcd^	84.3^abc^	61.8^ef^	53.1^f^	58.3^ef^				
Legumes	Dam	8.3^def^	15.6^bcd^	11.3^cdef^	4.9^ef^	7.1^def^	0.03	^***^	^***^	^***^
	Mixed	10.7^def^	11.1^def^	39.6^a^	40.5^a^	21.6^b^				
	Standard	1.60^f^	4.0^f^	14.9^bcd^	13.4^bcde^	21.0^bc^				
Forbs	Dam	5.3^cde^	8.8^cd^	7.3^cde^	3.2^de^	5.9^cde^	0.03	^***^	^**^	^***^
	Mixed	1.5^de^	2.1^de^	0.8^e^	6.8^cde^	11.9^c^				
	Standard	20.6^b^	11.8^c^	23.3^b^	33.5^a^	20.7^b^				
Tall	Dam	84.3^ab^	61.7^efg^	68.0^cdef^	76.8^abcd^	50.2^g^	0.06	ns	^***^	^***^
	Mixed	73.8^abcdef^	83.5^abc^	61.3^defg^	49.9^g^	52.2^g^				
	Standard	76.1^bcde^	89.2^a^	77.7^abc^	77.5^abc^	58.6^fg^				
Intermediate	Dam	7.0^g^	19.1^cde^	18.1^cdef^	11.9^efg^	32.3^ab^	0.04	^***^	^***^	^*^
	Mixed	14.0^defg^	11.9^efg^	34.8^ab^	26.4^bc^	41.5^a^				
	Standard	7.1^fg^	6.8^fg^	8.2^efg^	14.5^defg^	23.8^bcd^				
Short	Dam	6.7^cd^	10.4^bcd^	7.8^cd^	7.1^cd^	15.6^ab^	0.03	ns	^**^	^***^
	Mixed	7.4^bcd^	1.7^d^	3.9^d^	22.0^a^	6.3^cd^				
	Standard	2.9^d^	3.4^d^	13.4^abc^	5.9^d^	13.5^abc^				

*** p < 0.001;

** p < 0.01;

* *p < 0.05; + p < 0.10; ns p ≥ 0.10*.

a−g *Means within a variable with different superscript letters differ at p < 0.05*.

## Discussion

### Effect of Early Dam-Calf Contact and Grazing Experience on Calf Grazing Behavior

To our knowledge, only a few published studies have focused on dairy calf grazing behavior, and this is the first time that the effects of an early dam-calf contact have been investigated on calf grazing behavior, directly after weaning. As expected, the main differences in calf grazing behavior were mostly observed on the first day on pasture (Day 0): D-calves started grazing immediately when moved to pasture, whereas S- and M-calves started 23 and 43 min later, respectively. In several studies, it is reported that inexperienced heifers need a few hours (12) or a few days (24) to start grazing. This lag occurs even when animals are put on pasture with experienced heifers. In our study, the three groups were separated to prevent visual contact between experienced and non-experienced animals. Calves that had experienced pasture with dams in their early life then immediately remembered how to graze, unlike calves from the other groups. This is consistent with findings of Lopes et al. (24), who observed that heifers that had once experienced pasture instantly remembered how to graze the following year. The M- and S-calves took slightly longer to start to graze, probably because they had no social model or experienced individuals to emulate (33, 34). Also, inexperienced heifers spend more time exploring and tasting grass than ingesting it, compared to experienced heifers (33), which was numerically the case for our M- and S-calves here before they started grazing, even though exploring time was not long enough to statistically analyze it (data not shown). After starting grazing (on Day 0), M- and S-calves selected mainly “dry” patches, unlike D-calves, which directly grazed only “green” ones. This suggests that inexperienced calves could be neophobic (22): they were probably reluctant to try novel feed, and without a social model, were inclined to choose feed they already knew, or with similar characteristics to hay. Nevertheless, these differences were no longer seen in the following days, showing that calves can soon learn how to graze and cope with novelty.

Once they started grazing, all the calves followed the same pattern from Day 0 to Day 7: they first selected tall vegetation and then intermediate and short herbage as pasture utilization progressed. This is consistent with the selection of vegetation by stratum by experienced grazing cows under rotational grazing (35): once the upper layer is grazed, the height of the patch decreases, moving down to the lower layers (26). However, the botanical selection of the vegetation seemed different between groups: M- and S-calves ingested mainly grasses during the first days, as grasses are almost exclusive in the top layer, and then increasingly legumes and forbs [present in the intermediate and low layers, because of their smaller size; (34)]. On the contrary, D-calves constantly selected grasses until the seventh day of observation, whatever the height of the layer present on the plot. This suggests that inexperienced calves did not select vegetation according to its botanical composition, but rather ingested species according to their presence in the topmost layer as they utilized the plot. Calves that had experienced pasture with dams seem to have learnt to graze like adult cows, which are known to select grasses even on biodiverse pasture (26, 35). Furthermore, M- and S-calves tried to ingest *Rumex*, especially during the first day at pasture, while D-calves rarely approached it. *Rumex* is one of the main oxalate-producing plants: oxalate can cause poisoning in livestock if present in 10% or more of the dry weight of the plant (36). It is therefore important that cattle learn how to avoid it. This suggests again that calves that have grazed with their dams learnt to choose or avoid some plants (20), while inexperienced calves learnt by trial and error (21). Even though calves that did not experience grazing showed different grazing behavior than D-calves on the first day at pasture, their behavior evolved very quickly (less than a week) into behavior similar to adult cows. This implies that dam-calf contact close to birth has little impact on longer term grazing behavior.

Having experienced dam contact and/or pasture affected the time to start grazing and herbage selection behavior of dairy calves, but did not influence their daily ingestion time or the duration and number of their grazing cycles. All the groups of calves, regardless of their different previous experience, had the same grazing rhythm throughout the trial: this confirms that inexperienced animals exhibit similar grazing times to experienced animals, as found by Lopes et al. (24) and Hessle et al. (19). While idling, M-calves behaved differently from D-calves in *ad hoc* activities (i.e., walking, exploring, stereotypies and vocalizing). This was consistent with the finding of Arrazola et al. (33) highlighting that inexperienced calves spent more time walking and exploring compared to experienced calves, that spent more time inactive. Besides, M-calves spent less time lying than the calves in the other groups. Wilcox et al. (37) demonstrated that standing behavior could indicate a stress condition of the calves, especially in case of chronic stress. Even if we did not directly measure stress of the calves, it could not be excluded that repeating stress factors over time by splitting separation and weaning could have induced a stressful behavior for M-calves. We also found that M-calves spent less time ruminating than D-calves, while the latter spent more time resting. As rumination time is proportional to forage intake, this result suggests that although the ingestion time was similar between groups, M-calves may have ingested less forage than D-calves, as found by Arrazola et al. (33). However, the calves' daily forage intake was not monitored in the present study. A different digestibility of dry senescent and vegetative or tall and short patches (leaf to stem ratio) could also have affected rumination time, but the day by day differences among groups in patches characteristics are not consistent with the trend observed in rumination time. Furthermore, no differences in ADG between groups were observed before and after calves started grazing, even though in the literature inexperienced grazers were found to be nutritionally disadvantaged because of modest foraging behavior that could affect their live weight gains (19). This suggests that the calf daily forage intake was not different between groups. We cannot therefore confirm that the foraging skills of inexperienced calves were inferior, but we can assert that they were not typical of an adult cow.

### Effect of Early Dam-Calf Contact and Grazing Experience on Calves' Social Behavior

To the best of our knowledge, this is the first study to investigate dam-calf contact effects after weaning. Valníčková et al. (38) did not find any effect of dam-calf contact on social interactions or play behavior during colostrum feeding. Le Neindre and Sourd (39) found that heifers reared with foster cows dominated more than heifers reared without cow contact. We thus expected that calves reared with their dams would be more sociable or have more dominant behaviors than artificially reared ones, but we found no differences in negative interactions (i.e., dominance behaviors, such as head-butting, pushing, or fighting) between groups at pasture. Nevertheless, we observed that S-calves had more positive interactions with their companions (particularly licking) than did calves in the other two groups. Pinheiro Machado et al. (40) found that licking behavior between grazing dairy cows was not a random choice but showed a companion's preference for socio-positive interactions. Furthermore, they observed that licking was more persistent in long-established social groups. This could suggest that D- and M-calves may have created bonds rather with dams than with other calves, compared to S-calves, but this point requires further investigation. Besides, the higher proportion of time spent isolated by S-calves, compared to D- and M-calves, could suggest that they exhibit less gregarious behavior. It is however difficult to interpret, because of a lack of literature on this topic.

## Conclusion

Early life experience with dam and/or pasture influenced calves' foraging skills in the short term after weaning, especially on the first grazing day. Calves that had already experienced pasture with their dams immediately started to graze the day they were turned out to pasture in groups after being weaned. They instantly selected “green” patches of vegetation while grazing, unlike calves that had been housed indoors the whole time, which ingested predominantly senescent herbage on their first day. Daily ingestion time and duration and number of grazing cycles were not affected by previous experience. Nevertheless, botanical selection throughout pasture utilization and rejection of toxic plants (*Rumex*) showed that young calves could already exhibit post-weaning grazing behavior similar to that of adult cows when put on pasture early with their dam. This study provides evidence that separation of dairy calves from their dams close to birth has little impact on grazing behavior, as they grazed similarly to adult cows already in the short term (less than a week after being introduced to pasture). We spotted some differences in social behavior between the calves that experienced dam-calf contact and those that did not, but these differences are not easy to interpret and should be investigated in future studies. Further investigation is also needed to evaluate whether an early grazing experience with their dams could provide positive effects on behavior in the long term and performance in the future lactating careers of these calves.

## Data Availability Statement

The raw data supporting the conclusions of this article will be made available by the authors, without undue reservation.

## Ethics Statement

Ethical review and approval was not required for the animal study because the experiment was performed at Marcenat, INRAE experimental farm (Certificate of Authorization to Experiment on Living Animals No. D 15-114-01). No ethical approval was required because defined severity level was 0.

## Author Contributions

AN, MK, MC, MB, DP, and BM contributed to the conception and design of the study. AN, MK, MC, DP, and BM participated in the collection of behavioral observations data. AN and MK did the data curation and treatment. MK performed the statistical analysis. AN and MK wrote the first draft of the manuscript. MK and MC supervised the experiment and the writing of the manuscript. All authors contributed to manuscript revision, and all read and approved the submitted version.

## Conflict of Interest

The authors declare that the research was conducted in the absence of any commercial or financial relationships that could be construed as a potential conflict of interest.
